# Neotype designation for *Thymallus aeliani* Valenciennes, 1848 from a museum topotype specimen and its affiliation with Adriatic grayling on the basis of mitochondrial DNA

**DOI:** 10.3897/zookeys.999.56636

**Published:** 2020-11-30

**Authors:** Jernej Bravničar, Anja Palandačić, Simona Sušnik Bajec, Aleš Snoj

**Affiliations:** 1 Department of Animal Science, Biotechnical Faculty, University of Ljubljana, Jamnikarjeva 101, 1000 Ljubljana, Slovenia University of Ljubljana Ljubljana Slovenia; 2 First Zoological Department, Natural History Museum Vienna, Burgring 7, 1010 Wien, Austria Natural History Museum Vienna Vienna Austria

**Keywords:** Adriatic basin, control region, morphology, museum DNA, neotypification, taxonomy

## Abstract

In 1848, the grayling *Thymallus aeliani* (Valenciennes) was described from Lake Maggiore, Italy, in the north Adriatic basin. Genetic analyses of the mitochondrial control region showed a unique evolutionary history of grayling inhabiting the rivers of northern Adriatic basin, from the upper reaches of the Po River and its left tributaries in the west to the Soča River in the east, which resulted in the designation of this phylogenetic lineage as Adriatic grayling. Consequently, the name *T. aeliani* was connected to the Adriatic lineage, re-establishing the validity of this taxon. However, the mitochondrial haplotypes belonging to Adriatic grayling were never compared with the type specimens of *T. aeliani*, as their whereabouts were unknown. In this study, a neotype for *T. aeliani* was designated using topotypical specimens stored at the Natural History Museum in Vienna. The neotype (NMW 68027:2 labelled as “Lago Maggiore, Bellotti, 1880”) was designated pursuant to the conditions stipulated in Article 75.3 of the International Code of Zoological Nomenclature. Furthermore, the mitochondrial control region of the neotype was compared to haplotypes of the Adriatic lineage and showed high genetic similarity, which therefore connects the species name *T. aeliani* to the Adriatic grayling. This crucial step in fixing nomenclatural status of this species is very important for its protection and management.

## Introduction

The distribution of variation and disruption of the gene flow are multidimensional and continuous in nature; thus, specialists agree that delineating species can only be arbitrary (Galtier 2018). However, several research fields, such as ecology and macroevolution, and the general public need species as a simplified representation of natural variation. Ideally, biodiversity protection legislation would principally aim to protect management units (MU) or evolutionary significant units (ESU). Nevertheless, the European legislation focuses on endangered species, thereby making the species the basic unit of biodiversity protection. Specimens used to formally describe a species, called type specimens, are indispensable for determining the species affiliation of all subsequently analysed individuals. The absence of a type specimen can potentially be a significant source of nomenclatural instability. Accurate taxonomy is important for the identification of species and the evaluation of their conservation status, and without accurate identification, it is impossible to list those taxa whose existence is at risk and to set appropriate measures for their protection and management (Kottelat 1997).

An example of an endangered entity without a name-bearing type is Adriatic grayling, which represents endemic populations in the Adriatic basin, from the upper reaches of the Po River and its left tributaries in the west to the Soča (Isonzo) River in the east (Sušnik et al. 2001). Its clear distinctiveness from European grayling, *Thymallus thymallus* (Linnaeus, 1758), was recognised based on the mitochondrial (mt) control region (CR), revealing the Adriatic (AD) lineage that supposedly split from remaining European lineages about four million years ago (Sušnik et al. 2001; Meraner and Gandolfi 2012; Marić et al. 2012). Morphological studies of Adriatic populations from the mid-20^th^ century indicated differences between grayling from the Soča River (Adriatic Basin in Slovenia) and Sava River (Black Sea Basin) (Janković 1960, 1964), and distinguished Adriatic grayling from European grayling in the Danube, Ural, and Volga drainages (Bajić et al. 2018). However, especially in the study by Bajić et al. (2018), Adriatic grayling examined were introgressed with European grayling genome of domestic origin, and as such these comparisons should be considered with caution. Over the last 50 years, grayling from various European lineages has been and, in some parts, is still stocked into the natural range of the Adriatic grayling, resulting in introgressive hybridisation with native individuals (Sušnik et al. 1999, 2001; Meraner and Gandolfi 2012; Meraner et al. 2014). Introductions were also detected in the Ticino and Maggia rivers, the main inlets to Lake Maggiore, where Danubian mt haplotypes were detected (Sušnik et al. 2001).

In 1848, *Thymallus aeliani* (Valenciennes) was described on the basis of external morphology of grayling from Lake Maggiore (Po River drainage, Adriatic Basin) (Cuvier and Valenciennes 1848: 447–448). Later, this name appeared in the Catalogue of Fishes in the British Museum (Günther 1866: 201), though it was not included in Freshwater Fishes of the Austrian Monarchy (Heckel and Kner 1858), Siebold’s Freshwater Fishes of Middle Europe (Siebold 1863), and other important contemporary publications (e.g., Fatio 1882; Berg 1911). The name *T. aeliani* is also absent from subsequent reviews of the Italian fish fauna (e.g., Griffini 1903; Gridelli 1936; Tortonese 1970). The name reappeared in 1997 as a synonym of *T. thymallus* (Kottelat 1997) but was absent from the Handbook of European Freshwater Fishes (Kottelat and Freyhof 2007). The species *T. aeliani* was re-established by Bianco (2014), who considered the genetic distinctness, described by Sušnik et al. (2001), Meraner and Gandolfi (2012), and Marić et al. (2012) as sufficient to support species validity and connect the Adriatic lineage with the name *T. aeliani*. The species name was used as such, again without novel data, in two subsequent publications (Dyldin et al. 2017; Persat et al. 2019) and in the most recent checklist of Italian fish fauna (Lorenzoni et al. 2019). In their study to barcode (mt COI) circum-Mediterranean species, Geiger et al. (2014) deemed *T. aeliani* as a potential candidate species or a recovered synonym (appendix S1 in Geiger et al. 2014). In secondary sources, such as FishBase (Froese and Pauly 2010), *T. aeliani* is still listed as a synonym of *T. thymallus*. In the IUCN regional listing for Italy, Lista Rossa IUCN dei Vertebrati Italiani (Rondinini et al. 2013), Adriatic grayling is listed in the category of Endangered as *T. thymallus* pop. aut. The name *T. aeliani* has only recently been included on the IUCN Red List (Duchi et al. 2020), with deficient information indicating lack of knowledge of this species (e.g., species range).

At present, the whereabouts of the type specimens of *T. aeliani* are unknown, though they should presumably be deposited at National History Museum in Paris (see Discussion for more details). As such, it is not possible to objectively associate the species name referring to the type specimens from Lake Maggiore with the Adriatic grayling in its modern concept. Thus, neotypification of the Adriatic mt lineage provides the only solution if the name *T. aeliani* is to be tied indisputably to the Adriatic grayling. Owing to genetic mixing between native and introduced graylings mentioned above, extant populations are not proper candidates for the *T. aeliani* neotype selection, while no grayling translocations were recorded or discerned in the 19^th^ century (Povž 1995; Bianco and Ketmaier 2001; Povž and Šumer 2005). Therefore, museum collections from this period can offer suitable material to designate grayling neotype using specimens originating from the type locality (e.g. Splendiani et al. 2017). The ichthyology collection of the Natural History Museum in Vienna (NMW) houses grayling specimens from Lake Maggiore deposited in the museum in 1880 and 1881. We consider these specimens, originating prior to the translocations, as appropriate candidates for the neotype for *T. aeliani*.

The aim of this study was to designate a neotype for *T. aeliani* on the basis of 1) morphological comparison of the museum topotype specimens with the original description by Valenciennes (Cuvier and Valenciennes 1848: 447–448), and 2) sequenced CR of topotype specimens in comparison with the Adriatic mt lineage.

## Material and methods

### Material

To clarify the identity of *T. aeliani*, four white-eyed, museum topotype specimens registered under catalogue numbers NMW 68027:1–2, NMW 68090:1–2 were analysed and compared to the first description of the species, and then to the results of subsequent studies of the Adriatic grayling within the proposed species range. All four museum specimens are from the same time (1880–1881) and space (Lake Maggiore, type locality); the different naming is due to the inconsistent use of the name *T. aeliani*.

### Morphology

Measurements and counts (44 and 9, respectively) were taken of the topotype specimens of grayling deposited in the NMW ichthyology collection. Preserved specimens were examined under a stereomicroscope and photographs taken using a digital camera. Measurements were taken point-to-point using IP54 digital callipers with 0.1 mm precision. Relative measurements are presented as percentage of fork length (Lsm) or head length (HL). Morphological characters were compared to those listed in the original description of *T. aeliani* (Cuvier and Valenciennes 1848) and those applied in a morphological study of the Adriatic population from the Soča River (Adriatic Basin in Slovenia) (Janković 1960). According to the known history of stocking in Slovenia (Povž 1995; Povž and Šumer 2005), it can be speculated that the latter study was done on non-introgressed grayling.

### Molecular genetics

To link the species name *T. aeliani* to the Adriatic mt lineage of grayling (Adriatic grayling), we extracted DNA from the museum topotype specimens. Extra care was considered to avoid cross-contamination between specimens. Tissue for DNA extraction was taken from the right lateral fin, using sterilised and UV-irradiated utensils. Laboratory work was performed in a DNA clean room. For DNA extraction, QIAamp DNA Blood Mini Kit (Qiagen) was used, following the manufacturer’s protocol. All extractions included extraction controls to ensure there was no contamination of the buffers. DNA concentrations were quantified using a Qubit4 fluorometer (ThermoFisher Scientific, USA) and integrity checked on 1% agarose gel electrophoresis.

The complete mtCR was amplified using seven primer pairs (Table 2), designed based on the alignment of sequences found in European grayling to amplify overlapping fragments with lengths between 231–340 bp. The primers were designed using Primer3 (Untergasser et al. 2012).

All reactions were amplified using AmpliTaq Gold DNA polymerase (Applied Biosystems, USA) in 25 µl reactions according to manufacturer protocol with the use of an enhancer 360 GC, supplied by the manufacturer. Amplification was performed on a Veriti Thermal Cycler (Applied Biosystems, USA) using a simple two-step protocol with 10 min initial denaturation at 95 °C, 5 cycles of 30 sec denaturation at 95 °C, 30 sec annealing stage at 55 °C, and 30 sec elongation stage at 72 °C, followed by 40 cycles at 52 °C annealing temperature and final elongation stage of 7 min. Amplicons were checked for size on 1% agarose gel electrophoresis, purified with a Qiagen PCR purification kit and sequenced in both directions by LGC genomics (Berlin, Germany) using PCR primers. Sequences were checked visually and merged into a single sequence using BIOEDIT software (Hall et al. 2011) to construct the composed haplotypes of complete CR, which were subsequently compared to the NCBI database using BLAST.

### Phylogenetic analysis

The phylogenetic tree was constructed using Bayesian inference (BI) in BEAST 2.5.2 (Drummond et al. 2012). The obtained sequences were aligned with those from previous studies (GenBank accession numbers AF522395–AF522415, AF522418–AF522419, AF522425–AF522452; JX099337–JX099344, JX099346; JN796420–JN796435, JX144730–JX144732, KF280207–KF280208; Koskinen et al. 2002; Marić et al. 2012, 2014; Meraner and Gandolfi 2012) defining all known mt grayling lineages in Europe. Sequences of accession numbers AF522453 (*Thymallus arcticus*), AF522454 (*T. grubii*), and AF522455 (*T. brevirostris*) were used as an outgroup. The nucleotide substitution model was selected using hierarchical likelihood ratio tests implemented in MODELTEST 2.1.7 (Darriba et al. 2012). Three independent runs (50,000,000 steps) were performed and combined with LOGCOMBINER after 10% of each run was discharged as a burn-in phase.

## Results

### Morphology

**Figure 1.**
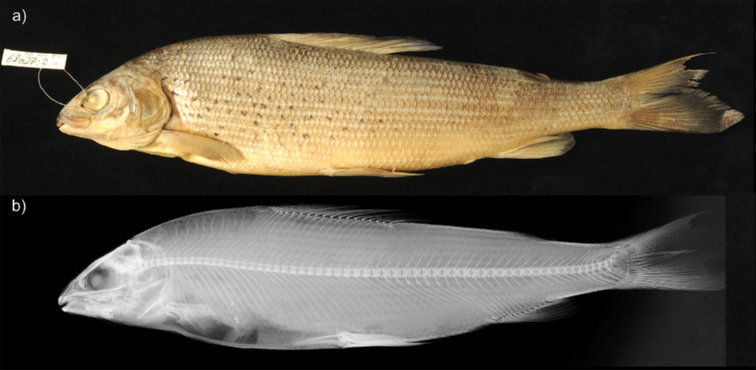
Photo (**a**) and radiograph (**b**) of the neotype *Thymallus aeliani* (NMW 68027:2) with fork length of 267.7 mm.

Measurements and counts are presented in Table 3. Low standard deviation (SD) values were observed in measurements when all museum specimens were treated as a single population, except for depth of the posterior part of the dorsal fin and head depth at the nape. Counts had the highest SD for the number of pyloric caeca and number of lateral line scales. The specimen NMW 68027:2 (Fig. 1), which matched with the original description of *T. aeliani* by Valenciennes and offered the best preservation of available specimens, was selected as the neotype. A comparative description of the neotype is provided in Tables 3, 4.

### Molecular genetics and phylogeny

DNA concentrations of isolates ranged from 25.8–29.4 ng/µl. Amplification and sequencing were successful in all four samples for each of the seven fragments. Length of overlapping fragments resulted in 1088 bp combined alignment of complete mtCR for each sample. No discrepancy in sequence between overlapping parts of fragments in any of the sample was observed, thus excluding possible contamination. A single haplotype was identified in all four specimens, and this sequence was deposited in the NCBI GenBank as *Thymallus aeliani* under accession number MT762347.

Alignment included CR sequences of grayling and the outgroup from GenBank and resulted in a total length of 1093 bp. Comparison of the sequence of topotype specimens to those in the NCBI database revealed that all four specimens carry the haplotype previously observed in grayling from the Adige and Adda Rivers and designated as Ad7 (GenBank acc. no. JN796420; Meraner and Gandolfi 2012). Furthermore, at least 98.5% identity to the haplotypes of the other Adriatic lineage was confirmed.

**Figure 2.**
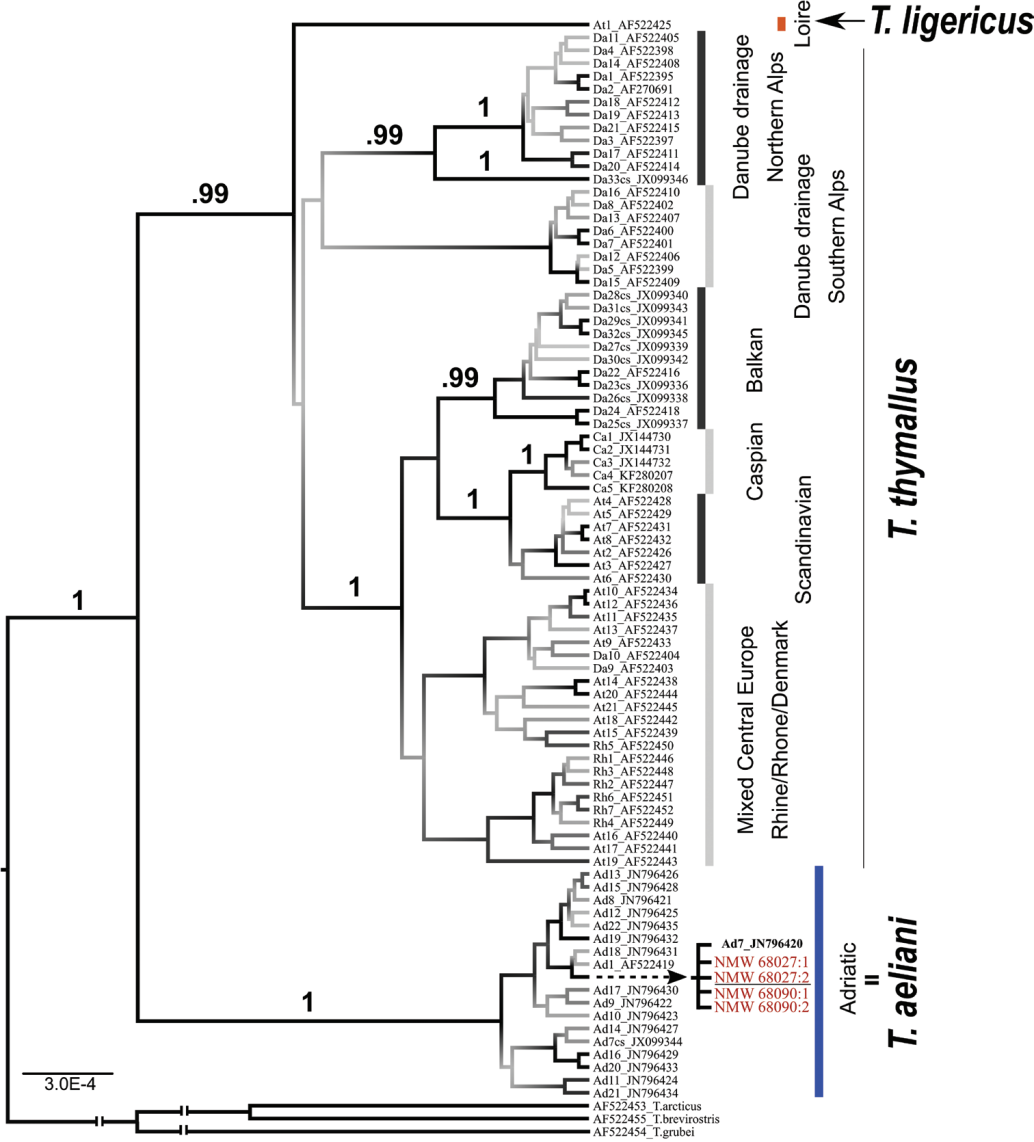
Phylogenetic tree with different evolutionary lineages of grayling found in Europe. Museum specimen haplotypes are shown in red, and the proposed neotype specimen is underlined. Posterior probabilities are shown above branches.

A phylogenetic Bayesian inference tree (Fig. 2), based on the complete CR sequences and constructed using the HKY+I +G nucleotide substitution model (Hasegawa et al. 1985), revealed that topotype specimens clustered together with other haplotypes endemic to the Adriatic basin with high support (posterior probability (pp) = 1). The Adriatic clade formed a sister clade to the monophyletic group of haplotypes found throughout Europe (*T. thymallus* + *T. ligericus*) comprising multiple previously described phylogenetic lineages.

## Discussion

Our findings regarding the fate of the syntypes for *T. aeliani* leading up to the neotypification for the Adriatic grayling are summarised below. The designation of the neotype, its morphological description and genetic identity of the neotype and the Adriatic mt lineage of grayling are discussed.

### Neotype designation

The main obstacle for the clear and indisputable clarification of the taxonomic position of *T. aeliani* was the inability to compare the Adriatic grayling with type specimens of the species. The syntypes of *T. aeliani* (in Cuvier and Valenciennes 1848) included three specimens of about equal length (ca 30 cm) collected by M. Savigny (Marie Jules César Lelorgne de Savigny) in Lake Maggiore (Cuvier and Valenciennes 1848: 447) likely prior to 1824 (Savigny ceased his professional activities by this year due to health issues; http://www.archives.seine-et-marne.fr/marie-jules-cesar-lelorgne-de-savigny-1777-1851). His specimens were deposited at the National Museum of Natural History (NMNH) in Paris, but to the extent of our knowledge, no other information was ever published about their existence, and according to the NMNH curators, these syntypes cannot be found in the museum and are considered lost (Dr Patrice Pruvost pers. comm, e-mail to AP of 04.09.2019). Since no type name-bearing specimens for *T. aeliani* are extant and the clarification of the taxonomic status of the Adriatic grayling is crucial for its conservation, we used museum topotype material from NMW in Vienna to designate the neotype.

Accordingly, a female specimen NMW 68027:2 (Lsm 267.7 mm, SL 252.5 mm) labelled “*T. vexillifer*, Lago Maggiore, Bellotti, 1880” is herein designated as the neotype under the conditions stipulated in Article 75.3 of the International Code of Zoological Nomenclature (ICZN) (International Commission on Zoological Nomenclature 1999). We failed to find an NMW accession book for this period, though the label text likely refers to an act of donation (or exchange) from Cristoforo Bellotti, honorary curator (*conservatore onorario*) at the Museo Civico di Storia Naturale in Milan at that time. Therefore, the actual date of collection is likely prior to 1880.

The morphology of the neotype fits the original description by Valenciennes (Cuvier and Valenciennes 1848: 447–448) (Tables 1, 2, Fig. 1). The diagnosis from the original description (Cuvier and Valenciennes 1848: 447–448) was based on the limited number of specimens and most of the listed characters are similar between *T. aeliani* and *T. thymallus*. However, the original description still provides a guideline for distinguishing *T. aeliani* from *T. thymallus* [as *T. vexillifer* in Cuvier and Valenciennes (1848: 438), unknown number of specimens originating from Lake Geneva and “northern Europe”]: short and shallow dorsal fin (vs long and deep), eight branchiostegal rays (vs 10), 84 total lateral-line scales (vs 87). The neotype corresponds to the original description both in number of branchiostegal rays and total lateral-line scales (Table 4). The distinguishing value of these characters was later supported by a detailed morphological study of an allegedly non-introgressed Adriatic grayling population from the Soča River (Janković 1960), although overlap is observed between the Adriatic and Danube populations in almost all characters (Table 4). All measured museum specimens in this study had eight branchiostegal rays, while the number of total lateral-line scales was variable: the specimens bearing catalogue number NMW 68027 had 83 total lateral-line scales, while specimens with catalogue number NMW 68090 had fewer scales (78 and 79). Three characters were considered of primary importance for diagnosing grayling populations in the Adriatic basin (Sabbadini 2000; Bianco 2014): (1) caudal fin colour in adults, which is reddish yellow to orange or red in non-Adriatic and blue in Adriatic populations, (2) a claret or pinkish stain above the pelvic fin, which is absent in the Adriatic populations, and (3) absence of the black spot on the throat in Adriatic grayling. However, the first two characters can only be evaluated in live, adult, spawning individuals, while the degree of colour expression is highly variable depending on habitat, season, sex, and size (Sabbadini 2000). Accordingly, these characters cannot be used to unambiguously identify live, young individuals or preserved collection specimens of any size. Nevertheless, the absence of the black spot on the throat was observed in all four museum specimens.

**Table 1.** Museum specimens used in this study.

Catalogue number	Designated name	Year of collection	Locality	Preservative
NMW 68027:1	*T. vexillifer*	1880	Lake Maggiore	Ethanol
NMW 68027:2	*T. vexillifer*	1880	Lake Maggiore	Ethanol
NMW 68090:1	*T. aeliani*	1881	Lake Maggiore	Ethanol
NMW 68090:2	*T. aeliani*	1881	Lake Maggiore	Ethanol

**Table 2.** Primers designed and used to sequence the complete mtCR of museum specimens. F: forward strand, R: reverse strand.

Fragment	Publication name	5–3´	F/R	Length (bp)
A	LRBT-25	AGAGCGCCGGTGTTGTAATC	F	267
	Thy_mus_A_rev	TGTGCTGATGTATGAGGGGT	R	
B	Thy_mus_B_for	CCTCTGACGCGCCTATGTTA	F	335
	Thy_mus_B_rev	TCGTTGGTCGGTTCTTACTACA	R	
C	Thy_mus_C_for	ACCCCTCATACATCAGCACA	F	338
	Thy_mus_C_rev	AGGTTAACCGCATCAACCAGA	R	
D	Thy_mus_D_for	AAGAACCGACCAACGATTTA	F	301
	Thy_mus_D_rev	TTCAAAGTTTAGTTCGACCTTATTAGT	R	
E	Thy_mus_E_for	CATGCATCTGGTTGATGCGG	F	340
	Thy_mus_E_rev	CGCGTAGAAGCCGGGGGA	R	
F	Thy_mus_F_for	AGAACTAATAAGGTCGAACTAAACT	F	231
	Thy_mus_F_rev	AGCGCTAATCGAGACTTCCTG	R	
G	Thy_mus_G_for	GAnTCCCCCGGCTTCTAC	F	306
	LRBT-1195	GCTAGCGGGACTTTCTAGGGTC	R	

**Table 3.** Measurements and counts of four historical NMW samples of *Thymallus* from Lake Maggiore, including the neotype of *Thymallus aeliani* – NMW 68027:2 in bold.

NMW number	68027:1	68027:2	68090:1	68090:2	Mean; SD
MEASURMENTS					
Lsm, mm (fork length)	282.6	267.7	289.9	282.3	280.6; 9.3
% *Lsm*					
body length to base of caudal fin	93.6	93.3	94.9	94.7	94.1; 0.8
trunk length	75.9	75.9	78.7	77.6	77; 1.4
preanal distance	68.6	69.5	70.5	70.6	69.8; 0.9
predorsal distance	33.1	36	33.1	34.2	34.1; 1.4
prepelvic distance	45.5	43.7	46.6	46.5	45.6; 1.4
distance between pectoral and pelvic fins	27.4	26.5	30	30.1	28.5; 1.8
distance between pelvic and anal fins	23.8	26.5	25.8	25.3	25.4; 1.1
length of pectoral fin	14	14.7	13.4	14.3	14.1; 0.6
length of pelvic fin	16	16	14	14.6	15.2; 1
length of base of dorsal fin	23	19.3	19.9	19.3	20.4; 1.8
depth of anterior part of dorsal fin	13	13.2	12.5	12.1	12.7; 0.5
depth of posterior part of dorsal fin	13.7	10.3	9.6	10.4	11; 1.8
length of base of anal fin	10.1	8.6	9.3	8.9	9.2; 0.7
depth of anal fin	12.2	12.3	11.8	12	12.1; 0.2
distance between anal fin and base of caudal fin	16.8	17.3	17	17.4	17.1; 0.3
distance between adipose fin and base of caudal fin	16.1	17.1	17.9	17.3	17.1; 0.8
length of caudal peduncle (as projection)	16.2	17.6	16.8	17.5	17; 0.7
body depth	20.6	22.2	24.3	21.2	22.1; 1.6
depth of caudal peduncle (minimum body depth)	7.1	7.6	7.3	7.8	7.5; 0.3
length of upper lobe of caudal fin	15.6	n/a	16	13	14.9; 1.6
length of lower lobe of caudal fin	n/a	18.1	n/a	15.7	16.9; 1.7
length of middle rays of caudal fin	5.7	6.8	5.7	6.4	6.2; 0.5
HL (head length)	18.8	18.9	18.7	19.3	18.9; 0.3
% *HL*					
snout length	5.8	5.4	5.8	5.9	5.7; 0.2
postorbital distance	5.8	5.4	5.8	5.9	5.7; 0.2
long diameter of eye	9.3	9.2	9.5	10.3	9.6; 0.5
length of maxillary	4.7	4.8	4.3	4.4	4.6; 0.2
depth of maxillary	6.7	6.2	6.2	6.8	6.5; 0.3
length of lower jaw	6.7	6.2	6.2	6.8	6.5; 0.3
interorbital width	1.9	2	2	2.3	2.1; 0.2
head depth at nape	8.1	8	8.2	8.9	8.3; 0.4
head depth through eye	5.3	5.3	5.6	5.8	5.5; 0.2
% *HL*					
snout length	30.9	28.7	30.8	30.4	30.2; 1
postorbital distance	49.3	48.9	50.6	53.3	50.5; 2
long diameter of eye	25	25.5	22.8	22.9	24.1; 1.4
length of maxillary	35.4	33.1	33.3	35.2	34.3; 1.2
depth of maxillary	10.2	10.5	10.9	11.9	10.9; 0.7
length of lower jaw	43.1	42.2	43.8	45.8	43.7; 1.5
interorbital width	28.4	28.3	29.7	29.9	29.1; 0.8
head depth at nape	69.5	68.7	73.1	74.2	71.4; 2.7
head depth through eye	51	50.3	49.5	50.4	50.3; 0.6
depth of posterior part of dorsal fin (% dorsal-fin base length)	59.8	53.4	48.2	53.9	53.8; 4.7
depth of maxillary (% length of maxillary)	28.7	31.7	32.6	33.9	31.7; 2.2
COUNTS					
total lateral-line scales	83	83	78	79	80.7; 2.6
total dorsal-fin rays	23.5	22.5	21.5	22.5	22.5; 0.8
branched pectoral-fin rays	13	14	13	13	13.3; 0.5
branched pelvic-fin rays	10	10	10	10	10; 0
total anal-fin rays	14.5	15.5	14.5	14.5	14.8; 0.5
gill rakers	22	23	n/a	21	22; 1
branchiostegal rays	8	8	8	8	8; 0
total vertebrae	59	58	59	57	58.3; 1
pyloric caeca	18	23	n/a	n/a	20.5; 3.5

**Table 4.** Characters distinguishing *Thymallus aeliani* and *Thymallus thymallus* (historic literature data compared to the neotype characteristics).

Character	Cuvier and Valenciennes (1848)		Janković (1960)		Sabbadini (2000)		Neotype of *T. aeliani*NMW 68027:2
Name in publication	*T. aeliani*	*T. vexillifer* (=*T. thymallus*)	*T. thymallus*, Soča River	*T. thymallus*, Danube tributaries	*T. thymallus*, Italy	*T. thymallus*, Danube	
Dorsal fin size	Short and shallow	Long and deep					
Depth of posterior part of dorsal fin (% dorsal-fin base length)			mean 43.0	means 50.7–56.3			53.4
Depth of posterior part of dorsal fin (% fork length)			5–14 [mean 9.4]	5–18 [means 11.0–12.2]			10.3
Head depth at nape (% head length)			62–82 [mean 71.7]	60–94 [means 73.1–76.4]			68.7
Number of branchiostegal rays	8	10					8
Number of gill rakers			20–25 [mode 21; mean 21.4]	(20, 21)22–29 [modes 24 and 25; means 24.7–25.3]			23
Number of simple rays in dorsal fin			7–10 [mean 8.1]	6–9 [means 7.0–7.3]			8
Total number of dorsal-fin rays			21–25 [mean 22.8]	20–24 [means 21.1–22.4]			22 [if two last rays counted as one ray]
Total number of vertebrae			57–61 [mode 59; mean 59.0]	55–62 [mode 59; means 59.0–59.4]			58
Number of pyloric caeca			18–38 [mean 26.6]	12–33 [means 18.0–20.3			23
Total number of lateral-line scales	84	87	78–92, most commonly 87–89 [mode 88; mean 86.8]	81–99, most commonly 88–92 [modes 88, 90 and 92; means 88.3–89.5]			83
Colour of caudal fin					Dark blue-grey	Reddish yellow to hot orange or red	
Black spot on each side of throat (under-part of mouth)					Absent	Present	Absent
Large magenta or claret blotch of irregular shape above and behind pelvic fin on both sides of body					Absent	Present	

### Genetic characteristics of the neotype

The neotype and three other specimens from the NMW historical fish collection listed in Table 1 were also molecularly defined by sequencing the mtCR. All museum specimens were found to share the same sequence, i.e. the Ad7 haplotype, which was previously confirmed in Adriatic grayling from the Adige and Adda Rivers (Italy), and this differed only up to 1.5% from other Adriatic sequences (Meraner and Gandolfi 2012). There are no data available on the recent frequency of native haplotypes in the Lake Maggiore drainage.

As seen in the phylogenetic tree (Fig. 2), the neotype sequence belongs to the monophyletic cluster that joins all CR sequences of grayling populations endemic to the northern Adriatic basin, and shows clear evolutionary distinctiveness from *T. thymallus* and *T. ligericus* (Persat et al. 2019). The clustering of CR sequences of the neotype and paratypes within the Adriatic mt lineage of grayling, connects the species name *T. aeliani* with Adriatic grayling in its modern concept. Therefore, based on the above, there can be no objection in applying this species name to the native grayling throughout the Adriatic basin.

The neotype designation for *T. aeliani* satisfies the provisions of Article 75.3 of the Code (ICZN) by: 1) clarifying the taxonomic identity of the Adriatic Grayling in its widely accepted modern concept (Article 75.3.1); 2) nominating its control region haplotype (GenBank acc. no. MT762347) as a diagnostic character (Article 75.3.2); 3) providing data and description sufficient to ensure recognition of the specimen designated (Article 75.3.3); 4) giving reasons and references for believing that original type material is lost (Article 75.3.4); 5) selection of the neotype is consistent with the original description of the species and collected not long after the original description and, as such, represent the native grayling that occurred in Lake Maggiore in the 19^th^ century (Article 75.3.5); 6) choosing a neotype from the originally cited type locality, Lake Maggiore (Po catchment, Italy) (Article 75.3.6); and 7) recording that the neotype is the property of a recognized scientific institution, NHM in Vienna (Article 75.3.7).
